# Altered Topological Patterns of Gray Matter Networks in Tinnitus: A Graph-Theoretical-Based Study

**DOI:** 10.3389/fnins.2020.00541

**Published:** 2020-05-27

**Authors:** Xiaofeng Lin, Yueyao Chen, Mingxia Wang, Chao Song, Bingling Lin, Xiaoping Yuan, Qingyu Liu, Haidi Yang, Ningyi Jiang

**Affiliations:** ^1^Department of Nuclear Medicine, Sun Yat-sen Memorial Hospital, Sun Yat-sen University, Guangzhou, China; ^2^Department of Nuclear Medicine, The Seventh Affiliated Hospital, Sun Yat-sen University, Shenzhen, China; ^3^Department of Radiology, Shenzhen Traditional Chinese Medicine Hospital, Shenzhen, China; ^4^Department of Hearing and Speech Sciences, Xinhua College of Sun Yat-sen University, Guangzhou, China; ^5^Department of Radiology, Sun Yat-sen Memorial Hospital, Sun Yat-sen University, Guangzhou, China; ^6^Department of Radiology, Peking University Shen Zhen Hospital, Shenzhen, China; ^7^Department of Otolaryngology, Sun Yat-sen Memorial Hospital, Sun Yat-sen University, Guangzhou, China

**Keywords:** tinnitus, structural analysis, graph theory, centrality, hub, edge

## Abstract

**Objective:**

Tinnitus is a prevalent hearing disorder, which could have a devastating impact on a patient’s life. Functional studies have revealed connectivity pattern changes in the tinnitus brains that suggested a change of network dynamics as well as topological organization. However, no studies have yet provided evidence for the topological network changes in the gray matter. In this research, we aim to use the graph-theoretical approach to investigate the changes of topology in the tinnitus brain using structural MRI data, which could provide insights into the underlying anatomical basis for the neural mechanism in generating phantom sounds.

**Methods:**

We collected 3D MRI images on 46 bilateral tinnitus patients and 46 age and gender-matched healthy controls. Brain networks were constructed with correlation matrices of the cortical thickness and subcortical volumes of 80 cortical/subcortical regions of interests. Global network properties were analyzed using local and global efficiency, clustering coefficient, and small-world coefficient, and regional network properties were evaluated using the betweenness coefficient for hub connectivity, and interregional correlations for edge properties. Between-group differences in cortical thickness and subcortical volumes were assessed using independent sample *t*-tests, and local efficiency, global efficiency, clustering coefficient, sigma, and interregional correlation were compared using non-parametric permutation tests.

**Results:**

Tinnitus was found to have increased global efficiency, local efficiency, and cluster coefficient, indicating generally heightened connectivity of the network. The small-world coefficient remained normal for tinnitus, indicating intact small-worldness. Betweenness centrality analysis showed that hubs in the amygdala and parahippocampus were only found for tinnitus but not controls. In contrast, hubs in the auditory cortex, insula, and thalamus were only found for controls but not tinnitus. Interregional correlation analysis further found in tinnitus enhanced connectivity between the auditory cortex and prefrontal lobe, and decreased connectivity of the insula with anterior cingulate gyrus and parahippocampus.

**Conclusion:**

These findings provided the first morphological evidence of altered topological organization of the brain networks in tinnitus. These alterations suggest that heightened efficiency of the brain network and altered auditory-limbic connection for tinnitus, which could be developed in compensation for the auditory deafferentation, leading to overcompensation and, ultimately, an emotional and cognitive burden.

## Introduction

Subjective tinnitus (henceforth referred to as tinnitus), also known as “ringing in the ears,” refers to the perception of sound in the absence of corresponding external source. It is a prevalent condition that affects approximately 10–15% of the adult population (Jastreboff, 1990; Heller, 2003; Shargorodsky et al., 2010), among whom an estimated 5–15% of the condition can become chronic and have a substantial negative impact on the quality of the patients’ lives (Dobie, 2003; Heller, 2003).

Current theories concerning the etiology of tinnitus mainly include a bottom-up deafferentation process following a hearing loss from cochlear damage, and a top–down maladaptive compensational mechanism (Rauschecker et al., 2010), resulting in hyperactivity in the auditory pathway (Eggermont and Roberts, 2004; Soleymani et al., 2011). A proposed model further suggested that the tinnitus sensation might be perceived only when aberrant neuronal activity in the primary auditory cortex is transmitted to a global workplace involving frontal, parietal, and limbic regions (De Ridder et al., 2011). The limbic system, with the thalamus, in particular, was proposed to play an inhibitory role in eliminating the noise signal transmitted to the global workplace (De Ridder et al., 2011). When the limbic regions become dysfunctional, noise-cancelation breaks down and the tinnitus signal pervades to the conscious perception (Rauschecker et al., 2010; Leaver et al., 2011). This model has been supported by empirical evidence from functional and structural imaging studies. For instance, using tract-based spatial statistics in diffusion tensor imaging data, one study found that the mean diffusion was significantly higher in the left superior, middle and inferior temporal white matter for tinnitus than control, which suggested enhanced connectivity between Heschl’s gyrus and limbic regions (Ryu et al., 2016). Furthermore, the compromised auditory representation could also be compensated with enhanced auditory memory retrieval through parahippocampal regions, thus sustaining the memory of the phantom sounds (Yoo et al., 2016b). These regions could constitute a complex network in maintaining and enhancing the symptoms, which is involved in not only auditory representation but also attention, memory, and emotion (Weisz et al., 2007; Leaver et al., 2011; Auerbach et al., 2014).

In recent years, connectome analysis of the brain has gained increasing popularity, given its ability in unraveling the complex network organization, and various network-based approaches such as graph-theoretical methods have been developed accordingly (Bullmore and Sporns, 2009; Lv et al., 2018). They have also been applied in tinnitus, which indeed helps find topological changes in their neural network properties (Mohan et al., 2016; Kandeepan et al., 2019). For instance, one study used a graph-theoretical approach to investigate the lagged phase functional connectivity in tinnitus using resting-state EEG and found that the topology of the patients’ network had increased regularity in low-frequency carrier oscillations, whereas decreased regularity in the high-frequency oscillations, which suggested maladaptive top-down modulation in compensation for the auditory deafferentation (Mohan et al., 2016). Another study using a graph-theoretical approach to investigate the relation between tinnitus distress and functional network activities found that tinnitus distress was strongly correlated within and between the right executive control network and the other four resting-state networks (Kandeepan et al., 2019). However, current topologic analyses on tinnitus neural networks have all been based on functional data. As far as we know, no studies have yet used topologic analysis approaches on structural data of tinnitus. Given that neural adaptation and age could lead to morphometric changes of the brain (Yoo et al., 2016b), it is likely that the topological changes seen in the functional neural networks of tinnitus could be accompanied with structural changes. Indeed, there has been structural evidence supporting changes in multiple brain regions in tinnitus (Mühlau et al., 2006; Simonetti and Oiticica, 2015), such as the Heschl’s gyrus (Schneider et al., 2009), and limbic regions such as thalamus (Mühlau et al., 2006) and parahippocampal cortex (Besteher et al., 2019).

In this study, we set out to explore network topology in tinnitus using structural MRI data with graph-theoretical techniques. We hypothesize that topologic characteristics could be seen in the structural networks of tinnitus, which is likely to involve both global and regional network changes, mainly auditory and limbic areas. Hence, we used several graph-theoretical based methods to assess the network properties of the brain, including global properties such as global efficiency (Eglob), local efficiency (Eloc), clustering coefficient (CC) and small-world coefficient (sigma), as well as regional properties including betweenness-centrality (BC) and interregional connectivity.

In graph theory, a network is defined as a set of nodes and the edges connecting them (Rubinov and Sporns, 2010; Bassett and Bullmore, 2017). Graph-theoretical studies have assessed structurally defined networks based on features such as gray matter volume, cortical thickness, surface area, and white matter connections between gray matter regions (Chen et al., 2008; Bassett and Bullmore, 2017). In the current study, a seed-based approach is adopted, where the nodes of a network correspond to anatomically segmented brain structures [regions of interest (ROI)], and the edges are defined by the strength of correlation between regional volume cortical thickness (Bassett and Bullmore, 2009; Rubinov and Sporns, 2010). Using these measurements, we assessed the topology of the brain networks by many properties, such as its functional segregation (CC and Eloc), functional integration (Eglob), small-worldness (sigma), centrality (BC) and edge (interregional connectivity analysis) (Rubinov and Sporns, 2010; Mijalkov et al., 2017). A more detailed introduction to these metrics can be found in Chap. 2.4.

## Materials and Methods

### Subjects

Tinnitus patients were recruited from Sun Yat-sen Memorial Hospital of Sun Yat-sen University in Guangzhou. In this study, we only recruited bilateral tinnitus patients, to avoid the possible confounding effect brought upon by laterality of the tinnitus. Inclusion criteria for the tinnitus group were as follows: (i) age between 18 and 60 years; (ii) all patients described their tinnitus as bilateral or originated within the head; (iii) tinnitus duration of more than 6 months; (iv) normal hearing level or mild hearing loss. To avoid confounding effects of severe hearing loss to the brain, hearing thresholds were controlled below 40 dB HL.

Exclusion criteria included objective tinnitus, Ménière’s disease, otosclerosis, chronic headache, severe alcoholism, smoking, head injury, stroke, Alzheimer’s disease, Parkinson’s disease, epilepsy, major depression or other neurological, psychiatric illness or major physical illnesses (e.g., cancer, anemia, and thyroid dysfunction). Moreover, we used the Hyperacusis Questionnaire to exclude participants with hyperacusis in the current study (Khalfa et al., 2002).

A total of forty-six patients with bilateral subjective tinnitus were eventually recruited for this study (all right-handed, 31 men and 15 women). Forty-six healthy subjects (all right-handed, 26 men and 20 women) with normal hearing were also recruited through a routine community physical examination and newspaper advertisements. All the subjects provided written informed consent before they participated in the study protocol, and the research was approved by The Research Ethics Committee of the Sun Yat-sen memorial hospital, Sun Yat-sen University.

### Audiological and Behavioral Assessments

All participants were screened for the extent of hearing loss using pure tone audiometry by the British Society of Audiology procedures at frequencies of 0.25, 0.5, 1, 2, 4, and 8 kHz (British Society of Audiology, 2012). Hearing thresholds were measured separately for the two ears. The hearing level for each ear was calculated as the numerical average of hearing thresholds of all the frequencies. Also, according to the self-rating depression scale (SDS) and the self-rating anxiety scale (SAS) (overall scores < 60, respectively), none of the participants had major depression or anxiety (Zung, 1971).

All tinnitus patients were interviewed to determine their tinnitus laterality and duration and for qualities of their tinnitus tone (pure tone-like tinnitus or noise-like tinnitus). Frequency and loudness of their perceived tinnitus tones were tested using audiometric tinnitus matching analysis. Depending on whether a patient perceives a pure tone or narrow-band noise, a pure tone or a narrow band noise at or around 1 kHz (1/3 of an octave above and below the center frequency) was presented contralateral to the (worse) tinnitus ear at 10 dB above their hearing threshold. The matching analysis was performed on the (better) tinnitus ear following the procedures recommended by the American Academy of Audiology (Henry and Meikle, 2000). The severity of tinnitus and related distress was assessed by the tinnitus handicap inventory (THI) (Newman et al., 1996) and visual analog scale (VAS) (Dawes and Haslock, 1982) (see Supplementary Table 1 for a sample).

### Image Acquisition and Preprocessing

We acquired structural T1 weighted MR images of all participants with a Philips 3-Tesla MR scanner (Achieva, Philips Medical Systems, Netherlands) with an 8-channel receiver array head coil. Structural images were acquired with a three-dimensional turbo fast echo (3D-TFE) T1WI sequence with high resolution (repetition time (TR) = 8.1 ms, echo time (TE) = 3.7 ms, slices = 170 (sagittal plane), thickness = 1 mm; gap = 0 mm; flip angle (FA) = 8°; acquisition matrix = 256 × 256; field of view (FOV) = 256 mm × 256 mm; and 1- mm isotropic resolution).

Image preprocessing was performed using the FreeSurfer software (version 5.3.0^1^) developed at the Martinos Center for Biomedical Imaging (Massachusetts General Hospital, Harvard Medical School) on a 64-bit Linux ubuntu 14.0. Preprocessing steps included removal of non-brain tissue using a watershed/surface deformation procedure (Ségonne et al., 2004), automated transformation to Tailarach space, intensity normalization (Sled et al., 1998), segmentation of subcortical gray/white matter tissue, tessellation of the gray matter – white matter boundary, automated correction of topology (Ségonne et al., 2007) and surface deformation (Fischl and Dale, 2000).

In the current study, we chose two measurements to construct the neural network: cortical thickness (CT) and subcortical volume. CT was chosen because it reflects the size, density, and arrangement of cells (neurons, neuroglia, and nerve fibers), which was found to show correlation with functional changes in development and disease (Narr et al., 2005; Chen et al., 2008; Bethlehem et al., 2017). This technique is known to be more robust than the widely used volumetry, as it is less sensitive to position errors and spatial variances (MacDonald et al., 2000) while also providing more precise measurements (Fischl and Dale, 2000; Pereira et al., 2012). Furthermore, subcortical areas could also contain important nodes that contribute to the generation of tinnitus (Rauschecker et al., 2010; Leaver et al., 2011), which cannot be captured by CT. Therefore, we used a combination of subcortical volumes with CT to construct a complete brain network. This method has been used by previous studies (Yun et al., 2020; Li et al., 2017). CT is defined as the Euclidean distance between the vertices of the gray-white surfaces (Singh et al., 2008), followed by parcelation of the cerebral cortex into 68 cortical areas (34 for each hemisphere) (Desikan et al., 2006). Subcortical volumetric analyses were performed using an automated procedure that estimates the probability of structure-classification based on prior templates in which those structures were manually identified (Fischl et al., 2002). We obtained 12 subcortical areas, including the thalamus, hippocampus, amygdala, caudate, putamen, and pallidum for each hemisphere. The anatomical labeling of the selected regions can be found in Supplementary Table 2.

### Network Construction and Assessment

Structural networks were constructed using GAT (Graph Analysis Toolbox) (Hosseini et al., 2012). The 68 cortical ROIs and 12 subcortical ROIs obtained in the last step were regarded as nodes in the current study. Regional cortical thickness and subcortical volume from these 80 ROIs were extracted and calculated for their correlation coefficients between subjects. After controlling for confounding effects of age and gender using linear regression, the residuals were used in the graph analysis. Possible edges were defined as the correlation of the cortical thickness and subcortical volumes between every pair of these nodes. The final structural brain network for each group contained a total of 80 nodes and 3,160 (80 × 79/2) possible edges. The definition of a valid edge was calculated with an above-threshold (absolute) correlation value that was set to 1, with an invalid edge with subthreshold values (including negative correlations) set to 0 (Gong and Xu, 2012), and this constituted an undirected (i.e., symmetric) and unweighted (binary) matrix.

Given that there could be discrepancies in the low-level correlation threshold between controls and patients’ brain networks (Yasuda et al., 2015), it is necessary to control the edge density (percentile of detected connections over the number of all possible connections). As there has been no established standard for a valid density, a range of different densities were used in this study. Specifically, edge density of the brain network was defined as the ratio of valid edges over total possible edges, i.e., 3160 edges, and in this study the density was controlled at a fixed range between 10 and 46%, to exclude disconnected networks and random network topology (Yasuda et al., 2015).

In this study, we used several methods to assess various global network properties of the brain, including global efficiency (Eglob), local efficiency (Eloc), clustering coefficient (CC), and small-world coefficient (sigma). Eglob and Eloc have been proposed to be able to reveal the network’s capability of integrating information effectively (Bullmore and Sporns, 2012). Eglob was used to assess the ability of a network to rapidly incorporate information from distinct anatomical regions based on the length of paths. A shorter length of paths indicated stronger functional integration, which was defined as the average of the inverse of the shortest path length between pairs of nodes (the smallest number of edges of which the path passes through any two nodes) in the entire network (Latora and Marchiori, 2001). Eloc, on the other hand, characterized the resistance of a network to failure on a small scale, by quantifying how well-information is exchanged by its neighbors when a node is removed. It was calculated from the average of the inverse characteristic path length among the neighboring nodes of the node (Bullmore and Sporns, 2012). CC was used to assess the presence of clustering within a network that indicates potential functional segregation, by computing the fractions of the nodes’ neighbors that were also neighbors to each other (Watts and Strogatz, 1998; Rubinov and Sporns, 2010). Finally, the small-worldness of a network, i.e., the ability of the network to reconcile the opposing demands of functional segregation and integration, was assessed with the small-world coefficient, i.e., sigma value, which was determined by the ratio of the clustering and the characteristic path length. Specifically, networks were considered to have small-world architecture if they had much higher clustering coefficients but an approximately equal characteristic path length when compared to a population of random networks with equal edge strength (Humphries and Gurney, 2008).

Regional network properties were assessed using normalized betweenness centrality (BC) and interregional correlations. The BC characterized the importance of a specific node, in which a node with high BC would indicate that it was part of “highly traveled paths.” In BC, the number of shortest paths that passed through a specific node was divided by the total number of short paths in the entire network (Brandes, 2001). In the current study, we considered a node to be a hub if its BC was at least 1.5 standard deviation higher than the mean network BC (Zhang et al., 2015). Interregional correlations were used to analyze edge properties between each pair of nodes. Previous studies have found that strong functional connections commonly exist between regions with no direct structural connection, and could be reflected a certain extent by the large-scale anatomical structure of the human cerebral cortex (Honey et al., 2009). Interregional structural correlations could capture these indirect structural correlations between two regions facilitated by a third party, from which diverse factors such as pathologic changes to the connectivity patterns could be detected (Evans, 2013). In this study, interregional correlations analysis was performed by directly comparing all the edge values (correlation coefficients) between the two groups.

### Statistical Analyses

Demographic and clinical data were analyzed using SPSS. 21 (IBM, Corp., Armonk, NY, United States). Demographic data were compared between the tinnitus and healthy control groups using independent-sample *t*-tests for continuous variables and non-parametric tests for categorical variables. Clinical data of the tinnitus group were summarized using descriptive analysis. Correlation between the cortical thickness/subcortical volume of the tinnitus patients and their clinical characteristics, such as average hearing threshold, SDS, SAS, the duration of tinnitus, THI, and VAS, was calculated using Pearson’s bivariate correlation and hierarchical regression analysis.

For the analyses of structural brain images, we used the GAT software. We first performed whole-brain comparisons of cortical thickness and subcortical volumes between the tinnitus and control groups. Before the comparison, cortical thickness data were first smoothed in surface-space using a 10 mm^2^ FWHM Gaussian kernel. Between-group comparisons (i.e., controls > patients) were then conducted vertex-wise across the entire cortex using independent sample *t*-tests, and results were thresholded at original *p* < 0.001, FDR corrected at *p* < 0.05.

For group-level network analyses, the four network property metrics (CC, Eglob, Eloc, and sigma) and interregional correlations were compared between the two groups. Specifically, metric values were subtracted for tinnitus > control and obtained a difference score for each metric. Non-parametric permutation testing was used to calculate the statistical significance of these difference scores. In each permutation, subject labels were randomly reassigned to 1 of 2 groups of the same size as the original groups, and the test statistics were calculated for each pair of randomized groups and subtracted in the same way as in the “real” data. The permutation was iterated 1000 times to form the distribution of difference scores from random data. Final results were thresholded at *p* < 0.001, FDR corrected at *p* < 0.05.

## Results

### Demographic and Clinical Data

Between the two groups, no difference was found in either the demographic (age, gender, and education years) or clinical status (hearing thresholds, SDS and SAS scores) (see Table 1 for more details).

**TABLE 1:** Demographic and clinical characteristics of study participants.

	**Tinnitus**	**Control**	**t/χ^2^**	***p*-Value**
**Age (year)**	40.9 ± 12.5	42.3 ± 14.2	0.503	0.608^a^
**Gender (M/F)**	31/15	26/20	1.153	0.283^b^
**Education (year)**	13.6 ± 3.9	14.0 ± 4.3	0.361	0.703^a^
**SDS score**	35.9 ± 11.3	31.9 ± 8.9	1.875	0.064^a^
**SAS score**	33.6 ± 13.4	29.6 ± 9.3	1.645	0.104^a^
**Hearing threshold (dB HL)**	20.02 ± 4.7	18.5 ± 3.0	1.866	0.065^a^
**Tinnitus duration (month)**	24.46 ± 12.31	NA		NA
**THI score**	54.59 ± 17.31	NA		NA
**VAS score**	2.97 ± 1.73	NA		NA

*SDS, self-rating depression scale; SAS, self-rating anxiety scale; THI, Tinnitus Handicap Inventory; VAS, visual analog scale; NA, not applicable. ^*a*^Two-sample *t*-tests. ^*b*^Chi-square test.*

### Whole-Brain Structural Difference Between the Two Groups

No significant difference was found for either CT or subcortical volumes between the two groups after FDR correction. No significant correlation was found between clinical data and CT/subcortical volumes.

### Global Network Properties

**FIGURE 1:**
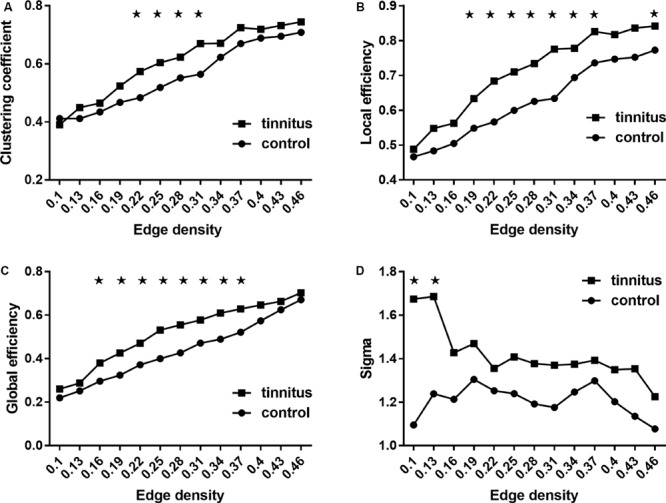
Between-group comparisons of global network measures. **(A)** Clustering coefficient, **(B)** local efficiency, **(C)** global efficiency, and **(D)** small-world property (sigma). **(A–C)** Increased as the network density increases, and **(D)** decreases as the network density increases. ★ Represents significant values. Results shown were thresholded at *p* < 0.001, corrected for multiple comparisons using the FDR procedure.

Figure 1 shows the analysis results of the global network measures in the two groups. CC, Eloc, and Eglob increased for both groups with increasing edge density values. At the same time, Sigma showed a generally similar pattern of small-worldness between the two groups, with fluctuations in tinnitus at density values of 0.1 and 0.13 (Figure 1). Group comparisons showed that tinnitus patients had significantly higher CC at network densities from 0.22 to 0.31, as well as increased Eloc and Eglob across most densities (from 0.16 to 0.37). Also, we found trends toward elevated small-world coefficients at a few network densities for the tinnitus group (Figure 1D). While the results of the small-world measures were rather unstable across densities, overall sigma values of the two groups were larger than 1, which indicated that the tinnitus brain maintained normal small-world characteristics with generally balanced global and local efficiency.

### Regional Network Analysis

**FIGURE 2:**
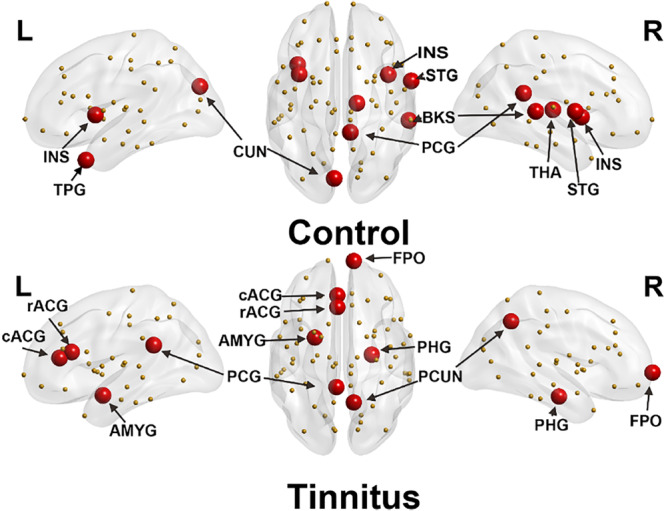
Distribution of hub regions of control (upper panel) and tinnitus (lower panel) based on regional betweenness centrality (mean + 1.5 SD). Large red spheres represent brain hubs and small yellow spheres represent non-hub brain regions. INS, insula; TPG, temporal pole; CUN, cuneus; STG, superior temporal gyrus; PCG, posterior cingulate gyrus; BKS, bankssts; THA, thalamus; rACG, rostral anterior cingulate gyrus; cACG, caudal anterior cingulate gyrus; AMYG, amygdala; PHG, parahippocampus; PCUN, precuneus; FPO, frontal pole; L, left hemisphere; R, right hemisphere.

The hub connectivity of the anatomic network was examined with BC separately for each group at an edge density of 22%. Eight hub regions were identified with high BC in the control group and seven in the tinnitus group (Table 2 and Figure 2). Three hubs with high BC in the superior temporal gyrus (STG), bilateral insula, and thalamus were only found for the control group. In contrast, three hubs in the amygdala and parahippocampus were only found for the tinnitus group.

**TABLE 2:** Betweenness centrality analysis for tinnitus and control.

**Group**	**Region**	**Normalized BC**
**Control**	Right-bankssts	5.940
	Left-cuneus	4.505
	Left-temporal pole	4.501
	Right-superior temporal	3.984
	Right-posterior cingulate	3.943
	Left-insula	3.905
	Right-thalamus	2.994
	Right-insula	2.926
**Tinnitus**	Left-caudal anterior cingulate	7.086
	Left-posterior cingulate	5.091
	Left-rostral anterior cingulate	3.336
	Left-amygdala	3.264
	Right-parahippocampal	3.263
	Right-precuneus	3.210
	Right-frontal pole	2.596

*Results shown were corrected at *p* < 0.05, FDR corrected. bankssts, banks of the superior temporal sulcus.*

**FIGURE 3:**
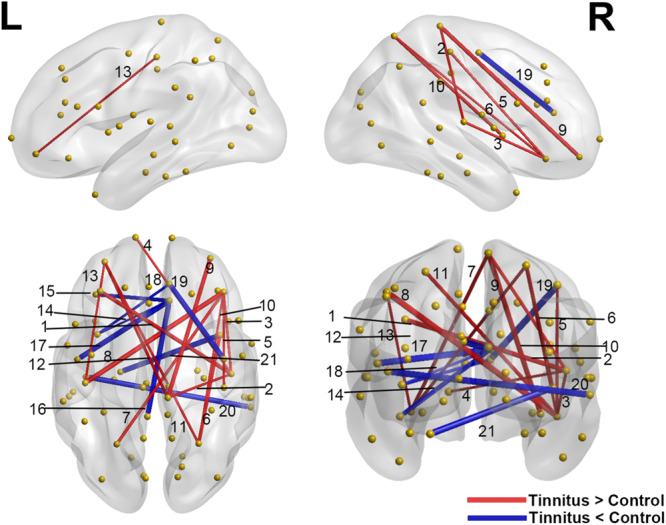
Significant differences of interregional correlations in the tinnitus vs. control comparison. Red lines indicate increased connection (tinnitus > control), and blue lines indicate the decreased connection (tinnitus < control). Yellow spheres represent non-hub brain regions, and the width of the lines correlate with the absolute values of the Z scores for the difference between two groups. L: left hemisphere; R: right hemisphere. Results shown were corrected at *p* < 0.001, corrected for multiple comparisons using the FDR procedure. The numbering of the lines corresponds to the connections listed in Table 3.

Interregional correlation analysis results were shown in Table 3 and Figure 3. Compared with controls, the tinnitus group showed generally higher connectivity between nodes, especially the right transverse temporal gyrus (the auditory cortex) to the prefrontal lobe (caudal middle frontal, right pars orbitalis, and left frontal pole) and the sensorimotor area (right paracentral). Meanwhile, the connectivity between the orbitofrontal cortex (OFC, including the right pars orbitalis, bilateral medial orbitofrontal gyrus) and the somatosensory area (bilateral post-central gyrus and superior parietal gyrus) increased. However, decreased connections were found between the right rostral- and caudal-anterior cingulate gyrus (ACG) with the left insula, pars opercularis, lateral orbitofrontal (OFC) and posterior cingulate gyrus (PCG), as well as between the left parahippocampus and right insula.

**TABLE 3:** Interregional correlation difference of tinnitus > control.

**Num**	**Interregional correlations**		**r(NC)**	**r(tinnitus)**	**z-Score**
**1**	**Right transverse temporal –**	Left caudal middle frontal	0	0.697	4.972^a^
**2**	**Right transverse temporal –**	Right paracentral	0	0.414	2.975
**3**	**Right transverse temporal –**	Right pars orbitalis	0	0.396	2.823
**4**	**Right transverse temporal –**	Left frontal pole	0	0.326	2.310
**5**	**Right pars orbitalis –**	Right post-central	0	0.399	2.828
**6**	**Right pars orbitalis –**	Right superior parietal	0	0.341	2.387
**7**	**Right pars orbitalis –**	Left superior parietal	0	0.504	3.585
**8**	**Right pars orbitalis –**	Left post-central	0	0.887	6.228
**9**	**Right medial orbitofrontal –**	Right paracentral	0.046	0.771	5.204
**10**	**Right superior parietal –**	Right insula	0	0.544	3.908
**11**	**Left medial orbitofrontal –**	Right superior parietal	0	0.42	3.300
**12**	**Left medial orbitofrontal –**	Right paracentral	0	0.489	3.506
**13**	**Left medial orbitofrontal –**	Left post-central	0	0.343	2.429
**14**	**Right rostral anterior cingulate –**	Left insula	0.308	0	−2.197^b^
**15**	**Right rostral anterior cingulate –**	Left lateral orbitofrontal	0.664	0	–4.784
**16**	**Right rostral anterior cingulate –**	Left posterior cingulate	0.932	0	–6.643
**17**	**Right rostral anterior cingulate –**	Left pars opercularis	0.977	0.006	–6.880
**18**	**Right caudal anterior cingulate –**	Left insula	0.424	0	–2.998
**19**	**Right caudal anterior cingulate –**	Right precentral	0.985	0.014	–6.797
**20**	**Left transverse temporal –**	Right middle temporal	0.971	0	–6.907
**21**	**Left parahippocampal –**	Right insula	0.997	0.013	–6.909

*NC, normal controls; r, Pearson correlation coefficient. ^*a*^Positive values indicate stronger connections for tinnitus than control. ^*b*^Negative values indicate weaker connections for tinnitus than control.*

## Discussion

In this study, we investigated the network topology changes in the gray matter of tinnitus patients. Multiple interesting results were found. First, the network topology of tinnitus exhibited increased CC, Eloc and Eglob, but maintained roughly similar small-worldness. Second, a different hub connection pattern was found for tinnitus that supports the existing theories on an auditory-limbic interaction. Specifically, compared with healthy controls, tinnitus patients were found with lower hub connectivity in the auditory cortex, insula and thalamus, and higher connectivity in the amygdala and parahippocampus. Finally, significantly altered interregional connections were found for tinnitus, including increased connectivity between the auditory cortex and PFC/sensorimotor region, and decreased connectivity between ACG and insula/OFC/PCG, as well as between insula and parahippocampus. To the best of our knowledge, this study is the first to investigate the topologic changes of structural network properties in the gray matter of tinnitus, and the results supported our hypothesis that altered topological morphological changes were present for tinnitus, which could underlie a maladaptive auditory-limbic interaction in the generation of their symptoms.

### Abnormal Global Network Properties in Tinnitus

The increased Eglob, Eloc, and CC found for tinnitus patients in our study suggest that the neural network of tinnitus has an abnormally higher network efficiency, with faster information flow across the brain (reflected by Eglob) and a higher level of local connectedness (reflected by the CC and Eloc). This could be a reflection of a compensational mechanism for the hearing loss (Langguth et al., 2013; Noreña and Farley, 2013). Specifically, the increased Eglob indicated a more compact global network, which could involve intensified information exchange from distributed regions. This could explain the common findings of increased connectivity between auditory and distress-related regions such as the amygdala (Rauschecker et al., 2010). On the other hand, an increased Eloc and CC could indicate an enhanced neuro-synchronization in localized regions, which has indeed been found for tinnitus in regions like the auditory cortex (Eggermont, 2006), and other regions, in particular limbic regions such as bilateral anterior insula, shown by a resting-state fMRI study using the regional homogeneity (ReHo) index (Chen et al., 2015b). Taken together, these findings indicated overcompensation in the structural networks of tinnitus, which could contribute to the development of distress, possibly through heightened local synchrony in auditory and limbic regions. On the other hand, no noticeable difference was found in the small-world property analysis between the two groups except for the two lowest densities. Both the neural networks in tinnitus and healthy controls in this study showed a small-world property (Mohan et al., 2016). This indicated a generally balanced modulation between integration and segregation was intact for the whole brain.

### Pathologic Changes in Hub Node Distribution of Tinnitus Patients

Highly interesting differences were found in hub connectivity measured by BC for tinnitus and the healthy controls, respectively. First of all, as compared to healthy controls, no hub was found in the primary auditory cortex in tinnitus patients. This anomaly is interesting because it has been proposed that hyperactivity in the auditory cortex could be a compensatory mechanism in tinnitus for the deafferented areas (Chen et al., 2015a). However, increased activity does not equate to increased integrity. Given that in BC, hubs are defined as nodes with high topological values, which are critical for efficient interactions, they are consequently associated with both integrative information processing and adaptive behaviors (Crossley et al., 2014). It could be that the hyperactivity within the auditory cortex prohibits this area from communicating with other regions of the brain, thus reducing the number of its edges and thus lowered BC, i.e., compromised network integrity.

Another finding is the absence of hubs in the thalamus for tinnitus. As reflected in the BC analysis for healthy controls, the thalamus serves as an important hub in the healthy population, exchanging information with multi-sensory pathways. It has been found to play an important role in sensory gating, i.e., selectively block certain unwanted sensory signals such as noise (Rauschecker et al., 2010). Based on our results, this hub characteristic of the thalamus is lost in tinnitus patients. This finding is strongly supported by several previous models indicating a dysfunctional auditory-thalamic interaction leading to a failed noise-canceling system (Rauschecker et al., 2010; Leaver et al., 2011).

Furthermore, high BCs were found in the insula for both sides of the brain in healthy controls while not in the tinnitus patients. The insula is proposed to be responsible for converging and integrating multi-modality sensory signals and assigning subjectivity into these signals, to form feelings and emotions (Craig, 2009; Menon and Uddin, 2010). In tinnitus patients, the lack of insula as a hub could be a result of the hyperactivity in the auditory cortex driving imbalanced attentional resources in various sensory pathways, i.e., excessive attention to the unwanted auditory signals, which could disrupt the regulatory ability of the insula and eventually lead to distress (Kandeepan et al., 2019).

Finally, the amygdala and parahippocampus were found with heightened BC in tinnitus, but not in healthy controls. The amygdala was found to be associated with especially negative emotions (Derntl et al., 2009; Feinstein et al., 2011), while the parahippocampal gyrus was found to be highly involved in memory (van Strien et al., 2009). The abnormally heightened connectivity of these two areas indicated a significant association for a negative emotion-memory system in these patients. In this system, the tinnitus perception could be associated with distress by primarily the amygdala and reinforced as a persistent awareness by the memory system through the parahippocampus.

### Changes in Interregional Connections in Tinnitus

Although we didn’t find hubs in the auditory cortex in tinnitus patients with the BC analysis, in the interregional connectivity analysis, we found enhanced connectivity of the right transverse temporal gyrus (Heschl’s gyrus) with especially bilateral frontal lobes. These frontal areas were primarily located at the lateral part of the cortex, adjacent to the dorsolateral prefrontal cortex (DLPFC). DLPFC is a crucial region in the executive control network that is proposed to be highly involved in cognitive control (Pessoa, 2014; Lehr et al., 2019), and has been used as an important target for stimulation in transcranial magnetic stimulation (TMS) and transcranial direct current stimulation (tDCS) treatment for tinnitus (Vanneste et al., 2011; De Ridder et al., 2012). Specifically, an EEG study showed that the responders to bihemispheric DLPFC tDCS differed from non-responders according to their resting brain activity in the right auditory cortex and parahippocampal area and the functional connectivity between DLPFC and the subgenual anterior cingulate cortex in particular (Vanneste et al., 2011). Combining our results and these previous findings, it is possible that there could be abnormal connections between the DLPFC and other areas of the brain, that could contribute to the distress of tinnitus, indicating a possible readaptation for the auditory loss/distress through cognitive modulation by strengthening connections with the frontal lobe.

Another interesting finding is the enhanced connectivity between the auditory cortex and the paracentral lobule in tinnitus. The paracentral lobule is adjacent to the somatosensory area (SMA), which was proposed to be involved in sensorimotor integration (Edwards et al., 2019; Umeda et al., 2019). Previous studies have found that some patients could modulate their tinnitus intensity by jaw protrusion (Lanting et al., 2010), suggesting enhanced auditory-somatosensory integration. Our finding provided structural evidence that the tinnitus brain may have an abnormally heightened exchange of auditory and somatosensory information. However, this assumption needs further testing with functional data.

Moreover, we found a decreased connectivity between the left parahippocampus and right insula. In the previous BC analysis, tinnitus was found with a lack of insula hubs and an additional hub in the parahippocampus. Combining the BC results with the current result, it shows a possible dissociation between these regions. Specifically, there could be generally compromised connectivity in the insula, resulting in the compromised modulating ability of multimodal sensory signals (Heydrich and Blanke, 2013). This change could result in a compensatory enhancement of the parahippocampal connectivity for the patients to retrieve auditory information from memory, which eventually leads to the sustaining of (phantom) auditory signals.

Finally, decreased connectivity between the ACG and left insula was also seen for the tinnitus patients. These are the two crucial nodes within the salience network (Menon and Uddin, 2010), which was proposed to assign saliency to various stimuli, and modulate dynamic interactions between various large-scale functional networks, such as the default mode network and executive control network (Uddin et al., 2017). This result is in slight disagreement with previous findings. In several previous studies, tinnitus has been proposed to have enhanced attention to especially tinnitus-related stimuli (Kandeepan et al., 2019) and was found to have more powerful activations in the salience network (Golm et al., 2013). One explanation for this discrepancy is that the enhanced functional activities found in the salience network might be a result of overcompensation from a diminished structural connection within the salience network. However, more research should be performed for this assumption.

The above findings are not only highly consistent with previous functional study findings, but they also provided empirical support for the anatomical basis for current theories of tinnitus. Current theories concerning the etiology of tinnitus mainly include a bottom–up deafferentation process following a hearing loss from cochlear damage, and a top–down maladaptive compensational mechanism (Rauschecker et al., 2010), resulting in hyperactivity in the auditory pathway (Eggermont and Roberts, 2004; Soleymani et al., 2011). A proposed model suggests that the tinnitus sensation might be perceived only when aberrant neuronal activity in the primary auditory cortex is transmitted to a global workplace involving frontal, parietal, and limbic regions (De Ridder et al., 2011), while the limbic system, in which the thalamus, in particular, plays an inhibitory role in eliminating the noise signal transmitted to global workplace. When the limbic regions become dysfunctional, noise-cancelation breaks down and the tinnitus signal pervades to the conscious perception (Rauschecker et al., 2010; Leaver et al., 2011).

### Limitation

Several limitations of this study should be addressed. Firstly, the structural brain networks were constructed based on inter-regional cortical thickness/subcortical volume correlations across subjects in groups of bilateral tinnitus patients and healthy controls. Each group could only form one structural matrix, and the individual matrix cannot be obtained. Therefore, only group-level analyses could be performed and could not provide individual correlations between brain structures and clinical data. This limited the power of the interpretations. It is desirable to obtain individual-level measurements of network properties using methods such as resting-state functional MRI and diffusion-weighted imaging data. Secondly, the chain of events and the causal interactions between brain networks cannot be inferred directly from structural data. Moreover, although anatomical covariance holds great promises in revealing the structural foundation for brain disorders, its exact relation with the various factors generating the diseases is still unclear (Evans, 2013). Finally, the small sample size is another limitation of this study. Future studies should be performed on patients with more diverse symptoms and larger sample sizes. For instance, in the current analyses, no correlation was found between any clinical characteristics (such as THI scores or SDS scores) of tinnitus and their structural parameters. However, previous studies have shown different results. For instance, it has been found that the cortical thickness changes related to hearing loss overlap with those related to normal aging, and tinnitus-related distress level and subjective loudness were found negatively correlated to the thalamic volume (Yoo et al., 2016a). Since tinnitus is also considered highly correlated with hearing loss, it could be that age and tinnitus severity are also correlated, which should both show a correlation with cortical thickness. Combining the above, results in this study should be interpreted with caution, and future studies investigating causal relations in the network dynamics are strongly suggested. For instance, resting-state fMRI could be combined with structural MRI to investigate the possible topographical changes in the functional network (Salvador et al., 2005; Achard et al., 2006), building a weighted network with multi-modality approaches such as fMRI and DTI (Jiao et al., 2020), or using combined volume-based and tract-based approaches (Husain et al., 2011) along with graph-theoretical analyses to investigate the causal relationship between duration/severity and brain network topology.

## Conclusion

In summary, by the construction of a brain network using cortical thickness and subcortical volume data, we have found alterations in the topology of structural brain networks of tinnitus. The heightened CC, Eloc and Eglob, indicated a generally more intensified information flow within both global and local properties of the tinnitus neural network, possibly as a compensatory mechanism for the deafferented signals. Subsequent regional analyses showed that hubs with high BC in the auditory cortex, insula, and thalamus were only present in the control group, but not in tinnitus. In contrast, hubs in the amygdala and parahippocampus were found for tinnitus but not in the control group. Interregional connectivity analysis showed enhanced connectivity between primarily the auditory cortex and prefrontal cortex/somatosensory areas, as well as reduced connectivity between primarily the insula and ACG/parahippocampus. Our findings provided the first anatomical evidence of gray matter network topological changes, supporting previous models of a dysfunctional auditory-limbic interaction that could be the underlying mechanism generating auditory phantom perceptions.

## Data Availability Statement

The datasets used and analyzed during the current study are available from the corresponding author upon reasonable request.

## Ethics Statement

The studies involving human participants were reviewed and approved by The Research Ethics Committee of the Sun Yat-sen Memorial Hospital, Sun Yat-sen University. The patients/participants provided their written informed consent to participate in this study.

## Author Contributions

XL and YC participated in experiment design, data collection and analysis, and writing of the manuscript. CS, BL, and XY participated in data collection and analysis. MW and QL participated in the discussion and revision of the manuscript. HY and NJ are the corresponding authors of this manuscript, who have full access to all the data in the study and take responsibility for the integrity of the data and the accuracy of the data analysis.

## Conflict of Interest

The authors declare that the research was conducted in the absence of any commercial or financial relationships that could be construed as a potential conflict of interest.

## Footnotes

1 http://surfer.nmr.mgh.harvard.edu
