# Supplementary material for: Altered Topological Patterns of Gray Matter Networks in Tinnitus: A Graph-Theoretical-Based Study
Source: Front Neurosci. 2020 May 27;14:541. doi: 10.3389/fnins.2020.00541 (PMC7267018; doi:10.3389/fnins.2020.00541)
Supplement: Supplementary file 1 [file Table_1.DOCX]

Supplementary Material

**Supplemental Table 1. Sample of the visual analogue scale**

| Items | VAS (10 for worst) |
| --- | --- |
| (1) The impact of tinnitus with/without hyperacusis on various everyday activites | 0-1-2-3-4-5-6-7-8-9-10 |
| (2) Percentage of time of being aware of tinnitus | 0-1-2-3-4-5-6-7-8-9-10 |
| (3) The degree of annoyance | 0-1-2-3-4-5-6-7-8-9-10 |
| (4) The impact of tinnitus on your life | 0-1-2-3-4-5-6-7-8-9-10 |
| (5) The intensity of tinnitus | 0-1-2-3-4-5-6-7-8-9-10 |
| (6) The level of distress caused by tinnitus | 0-1-2-3-4-5-6-7-8-9-10 |

**Supplemental Table 2. The cortical and subcortical regions of interest used in this study and their abbreviations.**

| (1,41) | Bank superior temporal | BKS |
| --- | --- | --- |
| (2,42) | Caudal anterior cingulate | cACG |
| (3,43) | Caudal middle frontal | CMF |
| (4,44) | Cuneus | CUN |
| (5,45) | Entorhinal | ENT |
| (6,46) | Fusiform | FFG |
| (7,47) | Inferior parietal | IPG |
| (8,48) | Inferior temporal | ITG |
| (9,49) | Isthmus cingulate | ICG |
| (10,50) | Lateral occipital | LOG |
| (11,51) | Lateral orbitofrontal | LOF |
| (12,52) | Lingual | LING |
| (13,53) | Medial orbitofrontal | MeORF |
| (14,54) | Middle temporal | MTG |
| (15,55) | Parahippocampal | PHG |
| (16,56) | Paracentral | PCL |
| (17,57) | Pars opercularis | Pper |
| (18,58) | Pars orbitalis | PaOR |
| (19,59) | Pars triangularis | PTR |
| (20,60) | Pericalcarine | PeC |
| (21,61) | Postcentral | PoCG |
| (22,62) | Posterior cingulate | PCG |
| (23,63) | Precentral | PreCG |
| (24,64) | **Precuneus** | PCUN |
| (25,65) | **Rostral anterior cingulate** | rACG |
| (26,66) | **Rostral middle frontal** | rMFG |
| (27,67) | **Superior frontal** | SFG |
| (28,68) | **Superior parietal** | SPG |
| (29,69) | **Superior temporal** | STG |
| (30,70) | **Supramarginal** | SMG |
| (31,71) | **Frontal pole** | FPO |
| (32,72) | **Temporal pole** | TPG |
| (33,73) | **Transverse temporal** | TTG |
| (34,74) | **Insula** | INS |
| (35,75) | Amygdala | AMYG |
| (36,76) | Caudate | CAU |
| (37,77) | Hippocampus | HIP |
| (38,78) | Pallidum | PAL |
| (39,79) | Putamen | PUT |
| (40,80) | Thalamus proper | THA |

**Note:** 1-40: left hemisphere, 41-80: right hemisphere.
